# Dealing With the Complexity of Effective Population Size in Conservation Practice

**DOI:** 10.1111/eva.70031

**Published:** 2024-12-13

**Authors:** Ancuta Fedorca, Joachim Mergeay, Adejoke O. Akinyele, Tamer Albayrak, Iris Biebach, Alice Brambilla, Pamela A. Burger, Elena Buzan, Ino Curik, Roberta Gargiulo, José A. Godoy, Santiago C. González‐Martínez, Christine Grossen, Myriam Heuertz, Sean Hoban, Jo Howard‐McCombe, Maria Kachamakova, Peter Klinga, Viktoria Köppä, Elenora Neugebauer, Ivan Paz‐Vinas, Peter B. Pearman, Laia Pérez‐Sorribes, Baruch Rinkevich, Isa‐Rita M. Russo, Adélaïde Theraroz, Nia E. Thomas, Marjana Westergren, Sven Winter, Linda Laikre, Alexander Kopatz

**Affiliations:** ^1^ Department of Wildlife National Institute for Research and Development in Forestry ‘Marin Dracea’ Brașov Romania; ^2^ Department of Silviculture, Faculty of Silviculture and Forest Engineering Transilvania University of Brașov Brașov Romania; ^3^ Research Institute for Nature and Forest Geraardsbergen Belgium; ^4^ Applied Population Genetics and Conservation Genomics, Department of Biology KU Leuven Leuven Belgium; ^5^ Department of Forest Production and Products University of Ibadan Ibadan Nigeria; ^6^ Department of Biology, Istiklal Yerleskesi Budur Mehmet Akif Ersoy University, Science and Art Faculty Burdur Türkiye; ^7^ Dokuz Eylül University Buca Education Faculty, Mathematics and Science Education, Biology Education Izmir Türkiye; ^8^ Department of Evolutionary Biology and Environmental Studies University of Zurich Zurich Switzerland; ^9^ Gran Paradiso National Park Alpine Wildlife Research Center Noasca Italy; ^10^ Research Institute of Wildlife Ecology University of Veterinary Medicine Vienna Vienna Austria; ^11^ University of Primorska Faculty of Mathematics, Natural Sciences and Information Technologies Koper Slovenia; ^12^ Faculty of Environmental Protection Velenje Slovenia; ^13^ Department of Animal Science University of Zagreb, Faculty of Agriculture Zagreb Croatia; ^14^ Institute of Animal Sciences Hungarian University of Agriculture and Life Sciences (MATE) Kaposvár Hungary; ^15^ Royal Botanic Gardens Richmond UK; ^16^ Department of Ecology and Evolution Estación Biológica de Doñana Seville Spain; ^17^ INRAE Univ. Bordeaux, BIOGECO Cestas France; ^18^ WSL Swiss Federal Research Institute Birmensdorf Switzerland; ^19^ The Center for Tree Science The Morton Arboretum Lisle Illinois USA; ^20^ The Committee on Evolutionary Biology The University of Chicago Chicago Illinois USA; ^21^ Royal Zoological Society of Scotland Edinburgh UK; ^22^ Institute of Biodiversity and Ecosystem Research at Bulgarian Academy of Sciences Sofia Bulgaria; ^23^ Technical University in Zvolen Zvolen Slovakia; ^24^ Czech University of Life Sciences Prague, Faculty of Forestry and Wood Sciences Department of Forest Ecology Suchdol Praha Czech Republic; ^25^ Department of Zoology Stockholm University Stockholm Sweden; ^26^ Behavioral Ecology Research Group Leipzig University Leipzig Germany; ^27^ Max‐Planck Institute for Evolutionary Anthropology Department of Human Behaviour, Ecology and Culture Deutscher Platz 6 Leipzig Germany; ^28^ Universite Claude Bernard Lyon 1 Villeurbanne France; ^29^ Department of Plant Biology and Ecology, Faculty of Sciences and Technology University of the Basque Country UPV/EHU Leioa Spain; ^30^ IKERBASQUE Basque Foundation for Science Bilbao Spain; ^31^ BC3 Basque Center for Climate Change Leioa Spain; ^32^ Israel Oceanographic and Limnological Research National Institute of Oceanography Haifa Israel; ^33^ School of Biosciences Cardiff University Cardiff UK; ^34^ Slovenian Forestry Institute Ljubljana Slovenia; ^35^ Senckenberg Biodiversity and Climate Research Centre Frankfurt Am Main Frankfurt Germany; ^36^ Norwegian Institute for Nature Research Trondheim Norway

**Keywords:** biodiversity monitoring, bridging science‐to‐application gap, effective number of breeders, genetic diversity, genetic indicators, Kunming‐Montreal global biodiversity framework, *Ne*, species conservation and management

## Abstract

Effective population size (*Ne*) is one of the most important parameters in evolutionary biology, as it is linked to the long‐term survival capability of species. Therefore, *Ne* greatly interests conservation geneticists, but it is also very relevant to policymakers, managers, and conservation practitioners. Molecular methods to estimate *Ne* rely on various assumptions, including no immigration, panmixia, random sampling, absence of spatial genetic structure, and/or mutation‐drift equilibrium. Species are, however, often characterized by fragmented populations under changing environmental conditions and anthropogenic pressure. Therefore, the estimation methods' assumptions are seldom addressed and rarely met, possibly leading to biased and inaccurate *Ne* estimates. To address the challenges associated with estimating *Ne* for conservation purposes, the COST Action 18134, Genomic Biodiversity Knowledge for Resilient Ecosystems (G‐BiKE), organized an international workshop that met in August 2022 in Brașov, Romania. The overarching goal was to operationalize the current knowledge of *Ne* estimation methods for conservation practitioners and decision‐makers. We set out to identify datasets to evaluate the sensitivity of *Ne* estimation methods to violations of underlying assumptions and to develop data analysis strategies that addressed pressing issues in biodiversity monitoring and conservation. Referring to a comprehensive body of scientific work on *Ne*, this meeting report is not intended to be exhaustive but rather to present approaches, workshop findings, and a collection of papers that serve as fruits of those efforts. We aimed to provide insights and opportunities to help bridge the gap between scientific research and conservation practice.

## 1 Applicable Methods for Estimating Effective Population Size Are Needed

Effective population size (*Ne*), defined as the size of an ideal population that experiences the same amount of genetic drift and increase of inbreeding as the real population (Wright 1931), is one of the most important parameters for assessing the long‐term viability of species and is, therefore, an important measure of conservation biology. The effective size of a population is essentially an evolutionary analogue to the census size (*Nc*), and it is a quantity that correlates to the loss or maintenance of genetic diversity and inbreeding within a population (Waples 2024a; Waples 2022). Higher *Ne* results in more maintenance of genetic diversity or lower levels of inbreeding and a faster response to natural selection and, thereby, adaptation to environmental changes. Hence, populations with higher *Ne* are expected to have higher survival probability. In 2022, due to the general acknowledgement of its importance for biodiversity conservation, *Ne* became the basis for a headline indicator for the monitoring and reporting of genetic diversity under the monitoring framework of the UN's Convention on Biological Diversity (CBD) Kunming‐Montreal Global Biodiversity Framework (GBF) (i.e., headline indicator A.4; CBD 2022; Hoban, da Silva, et al. 2024; Hoban, da Silva, et al. 2023). Consequently, effective population size is now embraced by governmental bodies and policy stakeholders, including national focal points for the CBD. In addition, effective population size has been included in relevant Essential Biodiversity Variables (EBV) for Genetic Composition (Hoban et al. 2022). Thus, practical and easy‐to‐use tools are needed to allow a diverse group of users to monitor and report progress on effective population size (Mastretta‐Yanes et al. 2024). However, choosing which method to apply and how to interpret the results is not straightforward. Given the different types of effective population sizes (Box 1), all referred to as *Ne*, the plethora of methods to calculate them, and the increasing number of different data sources available, we believe that there is a lack of scientifically evaluated and harmonized guidance for global, national, and regional reporting of *Ne*.

BOX 1 **What is *Ne*?**

Effective population size (*Ne*) is defined as the size of an ideal population that experiences the same amount of a given genetic property as the real population. In its purest sense, it assumes that a population is isolated and is at mutation‐drift equilibrium. There are many different types of *Ne* (inbreeding, variance, additive variance, eigenvalue, coalescence, metapopulation *Ne*), which are identical when the population is closed and at mutation‐drift equilibrium. We refer to Ryman, Laikre, and Hössjer (2019) for a comprehensive overview of how these differ when these conditions are not met. In the context of conservation genetics, the genetic properties ideally used to define *Ne* are additive variance *Ne*, allele frequency variance *Ne*, or inbreeding *Ne*, but other properties such as coalescence or linkage disequilibrium *Ne* can also be used. See our glossary (Box 2) for the definitions of these properties.

**FIGURE 1:**
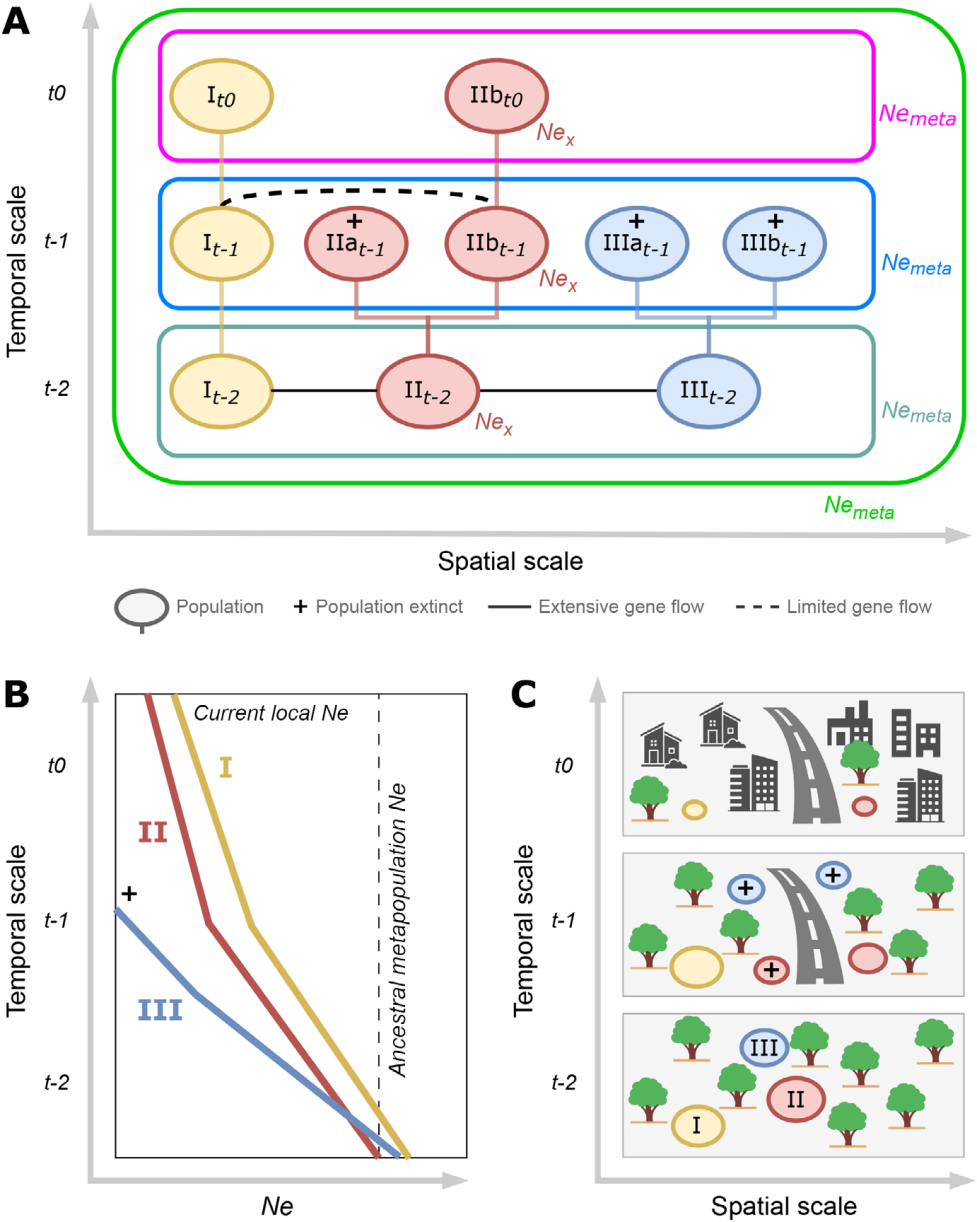
Schematic representation of a metapopulation evolving through time as an example, intended to highlight the possible ambiguity of *Ne* estimations. The X‐axis indicates populations' spatial distribution (sites) along one spatial dimension, for simplicity. As we go up and forward in time (Y‐axis), subpopulations disappear, and others are formed, but the metapopulation as a whole is maintained. (A) Sampling a single subpopulation (IIb) in the present (*t0*) and applying different *Ne* estimation methods may result in vastly different *Ne* estimates representing different aspects of the effective size, which are all commonly called “*Ne*”. Disambiguation of these different meanings is essential in conservation. Depending on the approach, one can estimate from the same sample local contemporary *Nex* (e.g., using linkage disequilibrium, kinship, or a temporal method), contemporary *Ne*Meta (when based on heterozygosity (*He*) decay across time; this requires two samples across time; pink), coalescent *Ne* (when based on its current *He*, and assuming mutation‐drift equilibrium; chartreuse), *Ne*Meta at different times in the past (blue, seagreen), but never past *Nex*. (B) Methods that estimate recent *Ne* trajectories (0–200 generations ago) will initially reflect local *Ne*, but will increasingly reflect metapopulation *Ne*, and samples taken in different subpopulations but with origins in the same metapopulation will eventually converge on the same *Ne*Meta, which is the sum of the past *Nex* (here at *t* *‐* *2*). The risk is that this is interpreted as a population decline, whereas it represents a confounding effect of spatial scale (Novo et al. 2023). (C) A landscape‐level schematic depiction of the processes occurring in (A) and (B).

Depending on how a particular study is designed, from sampling to the analysis method used, estimates of *Ne* from the same population can vary by orders of magnitude, not least because different types of *Ne* (Box 1) have different meanings in space (Ryman, Laikre, and Hössjer 2019; Waples 2024a) and time (Nadachowska‐Brzyska, Konczal, and Babik 2022; Tenesa et al. 2007) (see Figure 1). Different population properties affect *Ne* estimates, even when these converge to the same value under random sampling and certain ideal theoretical conditions, i.e., the population is isolated, of constant size, panmictic, has non‐overlapping generations, and is in mutation‐drift equilibrium (Ryman, Laikre, and Hössjer 2019; Waples 2024a). However, the lack of differentiation among different types of effective population size, often indistinguishably termed *Ne*, leads to confusion (e.g., Fady and Bozzano 2021; Hoban, Paz‐Vinas, et al. 2021).

The definition and, consequently, the value of *Ne* will also depend on the spatial extent of the target population and the sampling scheme. Populations can be isolated or connected by gene flow, forming metapopulations. When populations are not isolated, the administrative boundaries often do not coincide with the biological population, and it can be difficult to determine their size. Sampling schemes can involve several interconnected populations, a single population regularly sampled (connected or not with others), or a portion of a larger continuous population (Box 2).

BOX 2 **Glossary.**

**Additive variance**—Total effect on a trait stemming from one or more gene loci. **Census size (*Nc*)**—Number of individuals in a population that are reproductively mature. **Coalescence**—In population genetics, a model of how alleles sampled from a population may have originated from a common ancestor. **Demographic bottleneck**—an event that drastically reduces the census size of a population. **Effective population size (*Ne***)—Size of an ideal population that experiences the same rate of genetic drift as the observed population. **Genetic drift**—Random sampling of allelic variants that may lead to changes in the frequency of existing alleles from one generation to the next due to chance. **Isolation by distance (IBD)**—The decrease in the genetic similarity among individuals or populations as the geographic distance between them increases. **Life‐history traits**—A set of coevolved traits that affect an individual's survival and reproductive potential. **Linkage disequilibrium (LD)**—The nonrandom association of alleles at different loci. **Metapopulation**—In population genetics, a group of spatially separated populations of the same species that are connected by gene flow. **Mutation‐drift equilibrium**—The balance between new mutations introducing genetic diversity and random genetic drift removing (fixing) variants in a population. **Panmixia**—Random mating of individuals within a population that results in equal parental contributions to the next generation. Under Hardy–Weinberg assumptions, random mating occurs when allele frequencies accurately predict genotype frequencies.

On the temporal scale, *Ne* can refer to historical *Ne* or contemporary *Ne*. Historical *Ne* is a geometric mean of *Ne* per generation over many generations. As such, it explains the current genetic make‐up of a population and can be difficult to link with historical events, such as past management or anthropogenic environmental change. Contemporary *Ne* indicates the *Ne* of the current generation (or a few previous generations) and reflects the drift to be expected in the near future. This is of relevance for the ongoing monitoring of populations.

In real‐world conservation management, where formerly large populations have often become fragmented into small subpopulations, almost none of the underlying assumptions (i.e., isolation, panmixia, constant population size, etc.) are met (Ryman, Laikre, and Hössjer 2019). Thus, estimates of *Ne* obtained under realistic circumstances may be more or less inaccurate. Moreover, for practical conservation, *Ne* estimates may not be what nature managers, policymakers, and researchers believe they represent at the spatial and temporal scales. At the spatial scale, depending on how sampling was conducted relative to the actual population range and the method used, one could be estimating the *Ne* of a part of the population (subpopulation) or of the entire metapopulation (see Figure 1A and Ryman, Laikre, and Hössjer 2023; Waples 2010). When estimating *Ne* for conservation, it is essential to first determine the spatial scale of the ancestral and/or current metapopulation, as overlooking the importance of the spatial scale leads to dubious results (Clarke et al. 2024). As management units, sometimes determined by political boundaries, rarely correspond completely to biological populations, *Ne* estimates may not accurately reflect the status of the assessed populations and species, potentially misleading conservation planning, decisions, and actions. At the temporal scale, and depending on the estimation method, *Ne* estimates might reflect the historical *Ne* across several, up to hundreds of generations (i.e., coalescent *Ne*), or, when using methods for calculating contemporary *Ne*, the *Ne* estimate obtained might represent the *Ne* in the last two to three generations (Nadachowska‐Brzyska, Konczal, and Babik 2022).

Despite the pronounced scientific knowledge‐to‐application gap, genetic diversity concepts are increasingly being integrated into mainstream conservation, management, and biodiversity policy (Bertola et al. 2024; Hoban, Bruford, et al. 2021), where *Ne* remains a crucial summary statistic to evaluate the long‐term survival capacity of natural populations (Hoban, Paz‐Vinas, et al. 2021). Our article describes a workshop focused on refining methods for testing *Ne* using real‐world datasets. Attendees collaborated to address challenges such as data availability, missing data, and testing barriers across various taxa. The key outcome was to align conservation theory with practical challenges by estimating population sizes (*Nc*, *Ne*), focusing on species of conservation concern, and including diverse life histories and taxonomic groups to maximize conservation impacts.

### 1.1 Complexity and Reality of *Ne* Estimates

Over the past decade, the accessibility of DNA‐based and genetic monitoring has increased due to declining sequencing costs, wider availability of genomic data across many species, capacity‐building endeavors, and investments from international (e.g., EU), national, and private initiatives in conservation genomics (e.g., Earth Biogenome Project, EBP; International Barcode of Life, iBOL; or European Reference Genome Atlas, ERGA) (Theissinger et al. 2023). It has become practical and affordable to genotype individuals for tens of thousands of markers and estimate *Ne* with confidence intervals. However, numerous theoretical, technical, and methodological issues when estimating *Ne* need to be considered (Cox, Neyrinck, and Mergeay 2024; Gargiulo, Decroocq, et al. 2024; Mergeay et al. 2024; Pérez‐Sorribes et al. 2024; all in this Special Issue *Effective population size in conservation and biodiversity monitoring*) depending on the specific genetic markers, analysis methods, and sampling schemes.

Conservation genetic studies aimed at estimating allele frequencies have typically sampled 30–50 individuals, allowing the calculation of useful summary statistics (Allendorf 2017). When multiple sites are sampled, *F*‐statistics might be used to infer the genetic structure among subpopulations. *Ne* can be estimated from such samples using single‐sample estimator approaches (Jones and Wang 2010; Waples and Do 2010). These methods, however, can be sensitive to violations of the model assumptions, potentially leading to seemingly precise *Ne* estimates (i.e., with narrow confidence intervals) that may, however, be highly biased (Nunney 2016; Ryman, Laikre, and Hössjer 2023; Waples 2010).

Existing methods for *Ne* estimation vary widely in what they intend to estimate (*Ne* trajectories vs. point estimates; historical vs. contemporary *Ne*), the genetic signal they capture, and the data they require. Table 1 provides a brief description of the most commonly used methods, along with their main caveats. Most methods will assume neutrality and an isolated, random mating population with discrete generations. Population structure, in particular, biases *Ne* estimates obtained by many methods (Chikhi et al. 2010).

**TABLE 1:** Some methods commonly used to estimate effective population size *Ne*.

Estimation	Time scale	Type of method	Methods	Data required	Caveats	References
Effective population size (Ne) trajectories	Historical	Coalescent‐based	Skyline plots	DNA sequences	*Ne* reflects historical *Ne* of the total population. Sensitive to past population structure, which is often unknown.	Drummond et al. 2005; Ho and Shapiro 2011
	Historical	Sequentially Markovian Coalescent (SMC)	PSMC, MSMC	Single (PSMC) or multiple (MSMC) genomes sequenced to high coverage	Population structure can create spurious signals of population size change. Sampling regime can be important.	Li and Durbin 2011; Schiffels and Durbin 2014; Schiffels and Wang 2020
	Historical	Site frequency spectrum (SFS)	Stairway plot	Folded/unfolded SFS from hundreds of samples	May confound changes in gene flow and demographic changes.	Liu and Fu 2015, 2020
	Recent	Linkage Disequilibrium (LD)	GONE, LinkNe, SNeP	Phased or unphased genome‐wide genotype data	Will initially estimate local *Ne* and increasingly estimate metapopulation *Ne*, unless the population has always been closed.	Barbato et al. 2015; Hollenbeck, Portnoy, and Gold 2016; Santiago et al. 2020, 2024
Effective population size (Ne)	Long‐term	Site frequency spectrum (SFS)	dadi, fastsimcoal2	Folded/unfolded SFS from tens to hundreds of samples	May confound changes in gene flow and demographic changes.	Gutenkunst et al. 2009; Excoffier et al. 2013; Excoffier et al. 2021
	Contemporary	Linkage Disequilibrium (LD)	LDNe, NeEstimator V2	Genotype data from a single time‐point sample	Assumes independent markers, discrete generations, closed populations, and random mating. Very sensitive to spatial genetic structure.	Do et al. 2014; R. S. Waples and Do 2008
	Contemporary	Heterozygosity Excess	NeEstimator V2	Genotype data from a single time‐point sample	Assumes the only source of Hardy‐Weinbergdeviations is local inbreeding. Rarely accurate when *Ne* is not very small. To be avoided.	Balloux 2004; Do et al. 2014
	Contemporary	Temporal	NeEstimator V2; NB (R package), decay of heterozygosity over time	Genotype data from two or more samples separated by a known number of generations	Avoids confusing spatial and temporal genetic structure when sampling in metapopulations. May confound local and metapopulation *Ne* when migration rate is high.	Do et al. 2014; Hui, Brenas, and Burt 2021; Hui and Burt 2015; R. S. Waples 1989
	Contemporary	Sibship assignment	COLONY 2	Frequencies of full‐ and half‐sib dyads inferred from genotype data of single cohorts	Assumes discrete generations. Not sensitive to spatial genetic structure, but sensitive to the assumption of random sampling. Provides *Nb* of the sampled area.	Jones and Wang 2010; Wang 2009
	Contemporary	Approximate Bayesian Computation (ABC)	OneSAMP	Summary statistics obtained from genotype data	Computationally intensive when the number of loci is large. Estimates *Nex.*	Hong et al. 2024; Tallmon et al. 2008

For a more complete description of available methods and detailed accounts of their associated temporal aspects, see Nadachowska‐Brzyska, Konczal, and Babik (2022).

Here, we use the terminology of Ryman, Laikre, and Hössjer (2019) to designate specific aspects of *Ne*. We distinguish between the *Ne* of a subpopulation *x* in isolation (*Ne*x) and the realized *Ne* of subpopulation *x* when the joint effects of drift and gene flow are taken into account (*Ne*Rx), metapopulation *Ne* (*Ne*Meta), and coalescent *Ne* (*Ne*Co), based on frequently used methods for *Ne* estimation. Although important in their own way, we do not explore the differences among variance *Ne*, inbreeding *Ne*, gene diversity *Ne*, the effective number of breeders *Nb*, or additive variance *Ne* (Ryman, Laikre, and Hössjer 2019; Waples 2005). Notably, the common case of isolation by distance (IBD) in continuous populations can be considered as the ‘neighbourhood’ effective size (=*Ne*x of the neighbourhood) if the samples originate from a single neighbourhood (Neel et al. 2013; Cox, Neyrinck, and Mergeay 2024). *Ne*Co can be seen as the long‐term *Ne* that reflects the gene diversity under the assumption of mutation‐drift equilibrium.

To illustrate the ambiguity of these different types of *Ne*, a simplified example is provided in Figure 1A, where we consider a population consisting of two isolated subpopulations (I and IIb), with each subpopulation experiencing random mating. Suppose that subpopulation IIb is sampled at time *t0*; we could estimate *Ne*x from linkage disequilibrium (LD) (Waples and Do 2008) or sibship frequency (Jones and Wang 2010). With two samples from subpopulation IIb across a certain time span, we could also calculate (the variance) *Nex* using a temporal method, or at least the harmonic mean across the sampled time span. Under some circumstances, we could estimate *NeRx* using a temporal method that calculates the net genetic drift over that time span considering gene flow (Ryman, Laikre, and Hössjer 2019, but see Nunney 2016). Although at migration‐drift equilibrium, *Ne*Rx is similar across subpopulations and of the same magnitude as *Ne*Meta, *Ne* values obtained from the same subpopulation can easily be an order of magnitude different, yet they are all called “the effective size” (see Figure 1A–C).

It is possible to estimate the change in *Ne* over time for a specific sample using methods that trace back the historical trajectory of *Ne* over recent time (10s to 100s of generations) or ancient times (10^3^ to 10^5^ generations ago) (Nadachowska‐Brzyska, Konczal, and Babik 2022). When making inferences over time, we also need to acknowledge the limitations of changes in the spatial scale of reconstructions. While we sample an individual at time *t*, we also sample half of its parents' and a quarter of each of its grandparents' genes. We are also sampling across a wider geographic area as the spatial origins of ancestors of each individual widen the spatial scale due to gene flow across ancestral subpopulations. Therefore, when we sample a subpopulation and get an estimate of its *Ne* across time, it becomes increasingly difficult to interpret the *Ne* value if the population was not isolated across its entire history (Pérez‐Sorribes et al. 2024).

To illustrate that there is not a single measure of *Ne* that provides the definitive effective size of a population, we can consider the human population history. From a modern sample of human genomes, the genome‐wide nucleotide diversity reflects that our early ancestors underwent a prolonged bottleneck of about 1000 (effective) individuals (Hu et al. 2023). The long‐term coalescent *Ne*Co, which we can consider as the harmonic mean of the *Ne* of each generation, is still very low in the human population (tens of thousands) because of that bottleneck. However, if we were to calculate the contemporary *Ne*Meta of the global human population based on the variance in reproductive success, we would find *Ne*Meta to be around 3.8 billion. Both estimates of *Ne* are correct and useful, but they represent different concepts.

To visualize the challenges in estimating *Ne* in metapopulations, we can consider dynamic metapopulations (spatial variation) across time (temporal variation) to understand that some subpopulations could disappear as time progresses. Figure 1A,C provide schematic representations illustrating the dynamics of metapopulations and the challenges in estimating *Ne*. Depending on the methodology and sampling design, from a sample taken at *t0*, one could estimate the contemporary *Ne* of the metapopulation (*Ne*Meta) or the contemporary local *Ne* of a single subpopulation (*Nex*). But sampling population could also be used to estimate the ancestral (*t‐2*) *Ne*Meta. However, there are also countless ways model assumptions can be violated that can under‐ or overestimate the particular *Ne* of interest, especially when *Ne*′s different spatial and temporal types are confused.

When a *Ne* exceeds 500–1000 individuals, populations can generally maintain sufficient adaptive genetic variation (Frankham, Bradshaw, and Brook 2014). The 2022 Kunming‐Montreal Global Biodiversity Framework (GBF) has included specific goals and targets on safeguarding genetic diversity in the wild (Goals A and C; Targets 4 and 13) and specifically recognized that an effective size larger than 500 is required to maintain evolutionary potential (headline indicator A.4; CBD 2022). This criterion will likely seep through other biodiversity policy and management instruments (e.g., biodiversity strategies and action plans; Hoban, Hvilsom, et al. 2024), hence being of further significance in future conservation planning. This raises the question of both method and *Ne* type: what *Ne* should we focus on for present‐day conservation questions, and how can we best estimate it?

### 1.2 Operationalizing *Ne* Estimation for End Users: Bridging Science and Conservation Practice for Better Management of Genetic Diversity Within Species

Reflecting the extensive body of work dedicated to developing methods and tools for estimating *Ne* using simulations and empirical data (e.g., Frankham, Bradshaw, and Brook 2014; Gilbert and Whitlock 2015; Marandel et al. 2020; Nadachowska‐Brzyska, Konczal, and Babik 2022; Neel et al. 2013; Nunney 1999, 2016; Palstra and Ruzzante 2011; Ryman, Laikre, and Hössjer 2019; Tallmon, Luikart, and Beaumont 2004; Waples and Do 2010), to name a few, the members of Working Group 2 of the EU Cost Action G‐BiKE; (https://g‐bikegenetics.eu/en) organized a workshop to focus on the evaluation and implementation of *Ne* in biodiversity monitoring for better species management. A genetic diversity indicator that can use census population size (*Nc*) as a proxy for *Ne* (Hoban et al. 2020; Laikre et al. 2020) was recently adopted by CBD parties as a headline indicator A.4 for the monitoring framework of the Kunming‐Montreal GBF. Hoban et al. (2020) defined indicator 1 as “The number of populations within a species with an effective population size (*Ne*) above 500 compared to the number below 500” (headline indicator A.4 of the GBF, CBD 2022). When no direct estimate of *Ne* is available, typically due to a lack of genetic data, it is suggested to use *Nc* as a proxy, using as a rule of thumb an averaged *Ne/Nc* ratio of 0.10. Although this may be a conservative estimate (Clarke et al. 2024), the *Ne* > 500 threshold has been criticized for being overly liberal in some cases, rather requiring *Ne* > 1000 (Frankham, Bradshaw, and Brook 2014), especially in species with a low fecundity and therefore a high *Ne/Nc* ratio (Pérez‐Pereira et al. 2022). The proxy‐based methodology, using largely *Nc* or proxies of *Nc* to assess the CBD indicators, was developed elsewhere (Hoban, da Silva, et al. 2023; Mastretta‐Yanes et al. 2024; Hoban, da Silva, et al. 2024; Hoban, Hvilsom, et al. 2024).

**FIGURE 2:**
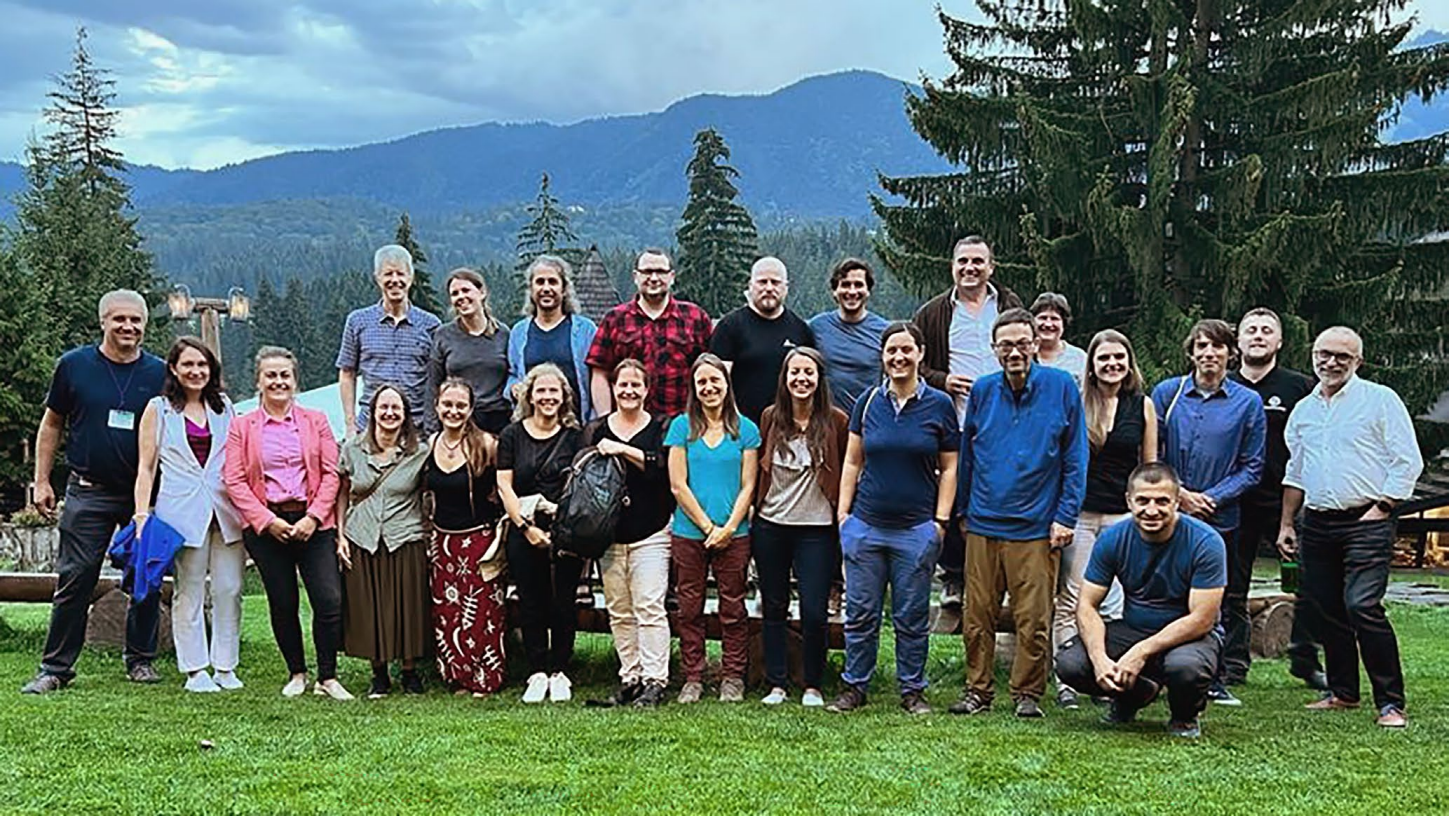
Participants of the international workshop, funded by the COST Action 18134, Genomic Biodiversity Knowledge for Resilient Ecosystems (G‐BiKE), August 2022 in Brașov, Romania. Participants who joined virtually are not shown.

In our hybrid meeting in Brașov, 26 experts from diverse European and international origins convened to discuss the pragmatic challenges of conservation implementing theoretical frameworks (Figure 2), and this was followed by numerous virtual meetings that included additional experts. Presentations at the workshop from attendees on the projects they were involved in included overviews of possible issues, available data, missing data, prospects, and potential barriers and solutions to *Ne* indicator estimation in their chosen taxa: Iberian lynx ( *Lynx pardinus* ), Wolverine ( *Gulo gulo* ), Grey wolf ( *Canis lupus* ), Alpine ibex ( *Capra ibex* ), European beech ( *Fagus sylvatica* ), maritime pine ( *Pinus pinaster* ), otters ( *Lutra lutra* ), and Wels catfish ( *Silurus glanis* ). We note that the *Ne* being referred to here is the short term/recent *Ne*, which determines the maintenance of genetic diversity, not the long‐term or coalescent *Ne*.

The central goal of the workshop was to conduct the groundwork to standardize protocols for the application of *Ne* estimators, with a focus on (1) determining the most robust methods across various scenarios and (2) elucidating the process of deriving a consensus *Ne* estimate for species of conservation significance, particularly as it was under the umbrella of EU Cost in the European context. The workshop evaluated widely distributed animal and plant species for which both census population size estimates and independent calibration of *Ne* estimates were available, laying the foundation for our analyses and evaluation. During the workshop, several challenges emerged in understanding *Ne* estimates. These included how to delineate populations across the continent and nations and establish the spatial scale at which *Ne* is to be estimated, how to best select methods based on the interplay between sampling design constraints, life‐history traits (social structure, dispersal capacity, overlapping generations, etc.), and methodological assumptions, and how we could gain better knowledge on these examples through forward simulations.

Addressing the challenge of defining populations involves balancing management considerations with biological and environmental factors that account for admixture, demographic and spatial expansion, but also ongoing fragmentation and range shifts of natural populations. The challenge of selecting the best tools and estimates involves consideration of temporal design (e.g., single vs. multiple events), sampling designs, and selecting appropriate approaches to estimate specific types of *Ne* while being aware of the assumptions and biases when assumptions are not met and of the type of *Ne* that is estimated with each approach.

For the effective size larger than the *Ne*500 criterion, the true *Ne* of interest is the additive variance *Ne*, which defines the rate of loss of additive genetic variance, or evolutionary potential (Ryman, Laikre, and Hössjer 2019). This is mostly a theoretical concept, but it is best approximated by the contemporary metapopulation (*Ne*Meta) or the realized inbreeding effective size (*Ne*Rx) of subpopulations within a metapopulation (Ryman, Laikre, and Hössjer 2019). In isolated populations, *Nex* and *NeRx* are evidently identical. In theory, some temporal methods (estimating *Ne* from the net change in gene diversity across a certain time interval) could estimate *NeRx*. In practice, this often depends on additional assumptions, and estimates can be hugely biased (Nunney 2016). Also, temporal methods that are based on measuring the variance in allele frequency across time will tend to yield a value close to *Nex* (Ryman, Laikre, and Hössjer 2019). In practice, we are often limited to methods that estimate *Nex*, whereas the metric of interest for the *Ne*500 threshold value is rather *Ne*Meta. As long as the absolute migration rate is low, the influence of gene flow on contemporary *Nex* is small. To estimate *Nex*, two methods are frequently used, and their sensitivities to assumptions and their performance (precision and accuracy in relation to sample size and the number of markers) have been tested extensively *in silico* (Do et al. 2014; Neel et al. 2013; Wang 2016; Waples 2021; Waples, Antao, and Luikart 2014). The sibship frequency method (Jones and Wang 2010) is not dependent on random mating and is rather insensitive to spatial genetic structure (Wang 2016). It requires random sampling and a good representation across the entire population distribution. However, when the true *Ne* is very large, it becomes imprecise unless the sample size is > 10% of the true *Ne*. The accuracy of the estimates obtained with the linkage‐disequilibrium method (Waples and Do 2008) is strongly dependent on spatial genetic structure and on the sampling strategy adopted; for instance, the method can provide unbiased estimates when local sampling is carried out in a subpopulation model unless the migration rate is high (Cox, Neyrinck, and Mergeay 2024; Neel et al. 2013). Mergeay et al. (2024) provide examples of this sensitivity compared to the sibship frequency method.

As long as metapopulations consist of well‐connected subpopulations (with one migrant per generation as a bare minimum for connectivity), we can, in theory, approximate *Ne*Meta by taking the sum of the *Nex* of all subpopulations (Cox, Neyrinck, and Mergeay 2024; Mergeay et al. 2024; Ryman, Laikre, and Hössjer 2019). Note that this approach is intended for an island model with symmetrical gene flow. In other cases (e.g., asymmetrical gene flow, very uneven subpopulation sizes, linear metapopulations, populations with extensive two‐dimensional isolation by distance, frequent extinction‐recolonization dynamics), more targeted models may be needed to estimate *Ne*Meta (Maruyama and Kimura 1980; Whitlock and Barton 1997; Nunney 1999). When gene flow falls below one migrant per generation, the correlation of allele frequencies among subpopulations becomes very weak, gene diversity within subpopulations rapidly decreases relative to the metapopulation, and inbreeding within subpopulations becomes increasingly important (Wright 1951). Consequently, it is of little use to estimate *Ne*Meta in a conservation context when gene flow drops below one migrant per generation.

Addressing taxon‐specific issues involves identifying mating systems, spatial structuring of populations, geographic distribution within countries, and availability of genetic data. We also tried to understand the relative influences of drift, inbreeding, and selection, which constitutes another major challenge. As such, *Ne* estimation tools used in conservation need to be forward compatible: we must ensure that they are easily integrated, among other things, with simulation approaches, species distribution modeling and climate change models (Haller and Messer 2019).

Tools such as GONE (Santiago et al. 2020) seem particularly relevant to reconstructing *Ne* changes that happened since the large‐scale influence of humans on biomes, ecosystems, and populations, as they provide a view on *Ne* in the recent past before we started monitoring biodiversity declines but after we started having a clear impact. Such tools complement methods that estimate the coalescent *Ne* or reconstruct long‐term *Ne* trajectories (Excoffier et al. 2013; Gutenkunst et al. 2009; Li and Durbin 2011; Liu and Fu 2015; Schiffels and Durbin 2014) and provide helpful insight into pre‐anthropogenic reference values of *Ne* or target values for ecosystem and population restoration in the long run. Even though GONE has been extensively tested *in silico* (Novo et al. 2023; Santiago et al. 2020) and even with experimental populations (Novo et al. 2023), empirical testing with real and often messy data, using legacy datasets to explore the benefits and limitations of the method, remains rare and needed (Gargiulo, Decroocq, et al. 2024).

**FIGURE 3:**
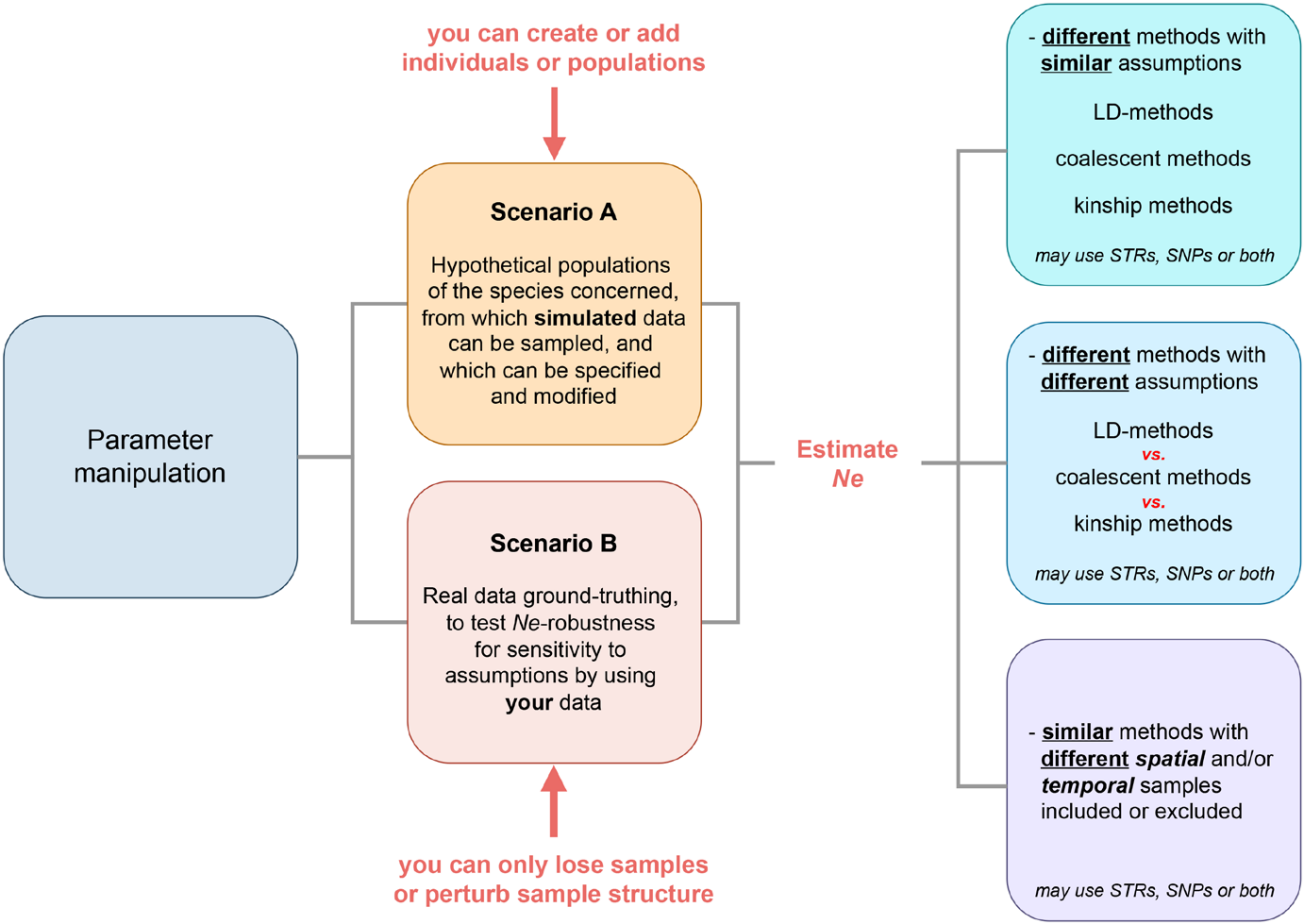
A schematic overview to evaluate various methods used to estimate the *Ne* by incorporating different techniques and underlying assumptions: The two approaches involved in parameter manipulation during the workshop were constructing hypothetical datasets using simulations to test a range of alternative scenarios on *Ne* estimation (scenario A) and manipulation of existing empirical datasets to mimic some of the likely biases that may occur (scenario B). Genetic markers such as short tandem repeats (STRs or microsatellites) or single nucleotide polymorphisms (SNPs) are used independently or in combination. *Ne* estimation involves different methods with divergent assumptions, including LD‐based approaches and kinship analysis. Similar to the first scenario (A), these methods may use STR, SNP, or a blend of both. Finally, *Ne* estimation strategies may involve consistent methodologies with variations in including or excluding spatial and/or temporal samples. These variations allow for an evaluation of how sample selection influences the accuracy of *Ne* estimation. These analyses may use STR, SNP, or a combination of genetic markers.

Two complementary approaches were proposed during the workshop (Figure 3): one focusing on constructing hypothetical datasets using simulations to test a range of alternative scenarios on *Ne* estimation where biases might exist (Figure 3, scenario A). The second approach was concerned with the manipulation of existing empirical datasets to mimic some of the likely biases that may occur and assess the effects of biases on the estimates (Figure 3, scenario B). The aim of these approaches was also to evaluate the performance of different software and how spatial and/or temporal scales affect *Ne* estimates.

In terms of parameter manipulation discussed during the workshop, our list is not exhaustive and could be modified based on the characteristics of the dataset. It is augmented by Hoban, Bertorelle, and Gaggiotti (2012), where additional information can also be found, including details on simulation applications, evaluation of simulator capabilities, and guidance for their use. Moreover, Hoban (2014) analyzed several case studies illustrating the use of simulations, elucidating their specific advantages and necessity, and exploring alternative or complementary (non‐simulation) approaches.

The suggestions raised during the workshop for parameter manipulation included *sample characterization* such as (i) *sample size*s that might be simply manipulated, for instance, by subsampling a reduced number of individuals: the size of the sample should be realistic as a function of *Nc*, and (ii) *sample distribution*, for example, different relevant sampling designs could be considered, depending on the population configuration and structure, including within meta, continuous, and isolated populations. An important aspect raised was how samples should be distributed based on what is known about dispersal and neighborhood size for the species (e.g., Cox, Neyrinck, and Mergeay 2024). Additional specific elements discussed were the *single temporal* sample estimates that can be compared with *multisample temporal* estimates where temporal sampling is available and, for the latter, different *times between sampling events*, for example, as a function of generation time, can be considered to compare *Ne* estimates across different timespans. Parameter manipulation requires consideration of *relatedness*; thus, keeping all relatives in the data versus pruning relatives can be done for estimations based on LD to ensure families do not dominate the dataset and *migration* (*Nm*, gene flow) is testing > 1 or < 1 (by moving genotypes from one population to another).

When DNA or genetic data is unavailable, as in many countries and regions (Pearman et al. 2024), proxy‐based indicator values of *Ne* are extremely important to evaluate and track multiple species affordably (Mastretta‐Yanes et al. 2024). Such proxy‐based indicators might, for instance, identify populations in urgent need of genetic monitoring and management (e.g., small and/or isolated populations), for which genetic data can be produced for a full assessment of genetic composition and change, such as small and/or isolated populations (Hoban, Paz‐Vinas, et al. 2024). A small population (in terms of census size) might be indicative of low effective population sizes, hence compromising the maintenance of genetic diversity over time by these populations. Once such populations are identified using affordable proxy‐based indicators, full genetic assessments needing DNA data production could preferentially target such populations to evaluate migration rates, inbreeding, the occurrence of genetic bottlenecks, etc. In this Special Issue of Evolutionary Applications, Mergeay et al. (2024) found a good correspondence between direct *Ne* estimates and *Nc* values for wolf populations in Europe. It is important, however, to study more in‐depth the relationship between *Ne* and proxies across species to identify situations where proxies serve their purpose but also where we need actual genetic data (Hoban, da Silva, et al. 2024). This approach can help us focus on species and populations where genetic and genomic resources must be developed.

### 1.3 Legacy Datasets to Test Method Suitability, Sampling Designs and Model Assumptions

We searched for so‐called legacy datasets: public or own genotypic data archives with sufficient metadata, large sample sizes, and, where possible, genotypic information from different genetic marker types. Especially large and spatially explicit sampling designs would allow us to test the importance of spatial sampling design, sample size, etc., which pertains to model assumptions of particular *Ne* estimation methods. A legacy dataset is a large, well‐annotated archived dataset that allows the calculation of specific properties of real populations, which can also be verified with independent data. In the context of population genetics, and especially of *Ne* estimations, these would be large genotypic or genomic datasets of real species and populations with well‐known properties associated with metadata of census size, age of individuals, possibly individual‐based spatial and ecological information or pedigree data, and data on reproduction and other life history traits (Pierson et al. 2018). Such datasets can be used to test the sensitivity of *Ne* estimation methods to underlying model assumptions, detect biases, and, by comparison, find what methodology and spatial sampling design best fit the known or expected *Ne*. It comes closest to simulated data, with the advantage of no dependence on the simulation assumptions. Analyses of such datasets can support the development of guidelines for sampling and analyzing species and populations with similar properties. A disadvantage of legacy datasets is that, unlike simulated data, the entire population is unlikely to be fully sampled, whereas simulated data can provide a complete sample.

In practice, only some legacy datasets fulfill all relevant criteria to carry out sensitivity analyses and test model assumptions. However, there are hundreds, if not thousands, of datasets that actually meet many or most of these criteria (Leigh et al. 2021), and which allow us to test aspects of model assumptions in *Ne* estimation. To develop standards for monitoring *Ne*, we sought to explore using legacy datasets to test the sensitivity of molecular *Ne* estimation methods to underlying model assumptions and optimize sampling designs for future projects. This Special Issue (*Effective population size in conservation and biodiversity monitoring*) presents papers that explore and analyze legacy datasets to better understand the sensitivity of *Ne* estimation methods to model assumptions (Cox, Neyrinck, and Mergeay 2024; Gargiulo, Decroocq, et al. 2024; Mergeay et al. 2024; Pérez‐Sorribes et al. 2024).

## 2 Working Group Discussions and Output

### 2.1 Challenges to *Ne* Estimation Differ Among Higher Taxa

**FIGURE 4:**
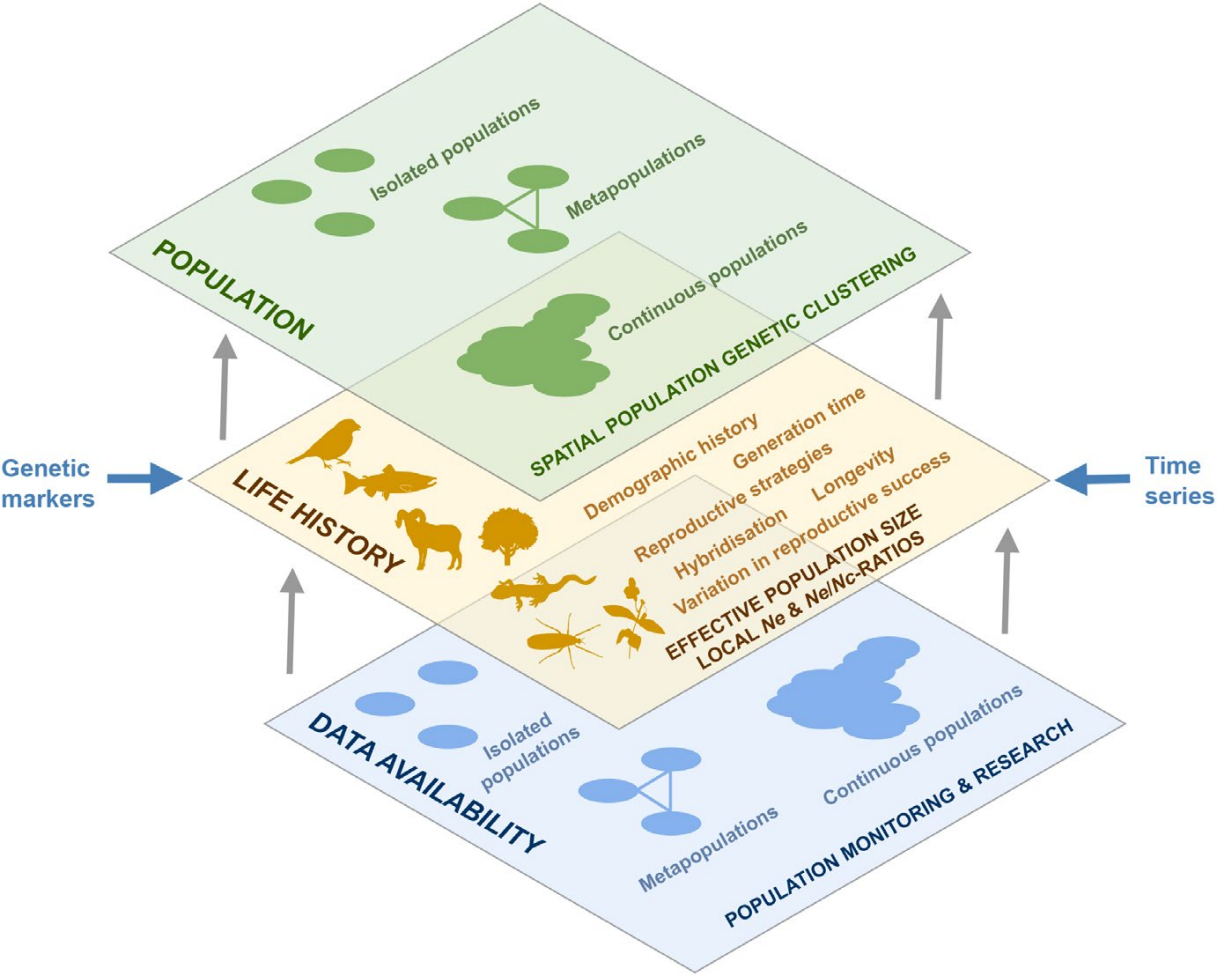
A general representation of the complexity of estimating effective population size (*Ne*). The figure presented is a conceptual model illustrating the complex framework used to assess genetic indicators in the context of conservation genetics. It details the interactions between the different levels of analysis and the factors that influence the assessment of genetic diversity in various species. At the highest level, ‘spatial genetic clustering of populations’ is highlighted, distinguishing between isolated populations, metapopulations, and continuous populations. This classification is key to understanding gene flow and genetic structure, which are fundamental to conservation strategies. At the middle level, the “life history level” includes aspects such as demographic history, generation time, reproductive strategies, differences in reproductive success, and hybridization. These elements influence the effective population size and the *Ne/Nc* ratio, which are essential parameters in the study of population genetics; this life‐history data primarily provides insights into local *Ne*. Genetic markers and time‐series data are essential for comprehending the structure and dynamics of populations at a spatial scale, yet they have the potential to introduce biases that could diminish the effectiveness of the analysis. The framework underscores the necessity of synthesizing data from genetic markers with an in‐depth understanding of species' life histories. Additionally, it highlights the imperative to secure the availability of extensive genetic data, which is critical to informing effective conservation strategies.

Specific working groups discussed factors that affect *Ne* estimations in species of animals (reptiles, amphibians, fish, and mammals) and plants, and the available datasets were evaluated. The discussions also helped identify the common factors influencing *Ne* estimates across taxa. The potential influential factors on the estimation of *Ne* are reflected and summarized in a multi‐layer schematic that represents aspects of the available data, species life histories, and population characteristics (Figure 4). Finally, the discussions helped working groups to identify datasets that have served as the basis for research that is reported in this Special Issue and elsewhere.

### 2.2 Population Structure: Continuous Distribution, Isolation‐By‐Distance, and Recent Expansions

Accurate identification of populations and population structure may impact *Ne* estimation and arise in multiple higher taxa. Recent population recovery has resulted in the re‐establishment of connectivity between previously isolated, genetically differentiated units; what appears to be a spatially contiguous population may have significant spatial genetic structuring and violate the assumption of a single panmictic population. Some carnivores and larger mammals in Europe have experienced recent population recoveries from small isolated populations yet may still exhibit substantial spatial genetic structuring (Thomas et al. 2022). Challenges emerged from complexity in defining populations for accurate *Ne* estimation, especially for mammal species exhibiting hybridization (Adavoudi and Pilot 2021) and population structure and continuous species distributions (Adavoudi and Pilot 2021; Iacolina et al. 2021; Randi 2007). Identifying genetic boundaries among populations is important for determining appropriate sampling strategies to avoid pooling populations. In contrast, widely distributed species of plants may experience isolation by distance (IBD) and absence of panmixia, which may be difficult to compensate for with any sampling strategy. Similarly, *Ne* estimation for fish and other riverine species populations is likely complicated by spatial solid patterns of genetic variation, isolation by distance, and potentially metapopulation dynamics. The influence of population traits on *Ne*, particularly differences in demographic stochasticity and reproductive variance, requires further investigation (May et al. 2023; Wright, Schofield, and Mathews 2021).

Different characteristics affecting *Ne* estimation were identified for fish, depending on whether the species is a cartilaginous or bony fish species and the species' main habitat (marine, rivers, lakes, and ponds). Difficulties in *Ne* estimation arising from the typically large *Nc* and *Ne* and associated stochasticity observed for marine fishes have been discussed previously (e.g., Marandel et al. 2019; Montes et al. 2016). However, cartilaginous fish like rays and sharks, while mainly marine, seem not to display *Ne* values as high as those of bony marine fishes (Hoban et al. 2020), and the potential effects of the low reproductive output and high longevity of cartilaginous fishes compared to bony marine fishes were discussed. *Ne* variation within and across taxonomic groups has recently been addressed (Hoban, da Silva, et al. 2024). A talk during the workshop further addressed technical limitations related to estimating *Ne* and *Nc* in a large‐bodied fish species ( *Silurus glanis* ) that inhabits large river systems and has undergone a demographic expansion (Paz‐Vinas et al. 2024). Finally, the determination of population extent is further complicated by hybridization between species. For instance, for amphibians, the groups identified the potential importance of hybridization following secondary contact (e.g., for salamanders, Bruni et al. 2023; Patton et al. 2020). Hybridization could potentially affect *Ne* estimation in many plant species.

In contrast, the group discussions recognized population characteristics that may facilitate *Ne* estimation when population definition or extent is relatively easy to define. The strong spatial structure, genetic isolation, and clear population boundaries of many reptile species and pond‐breeding amphibians were highlighted as factors that might facilitate *Ne* estimation, as these characteristics of populations may approximate the many assumptions made by most *Ne* estimation methods. In fish species inhabiting isolated lakes and ponds, conformity to some assumptions of the Wright‐Fisher model (i.e., no immigration, constant population size) might be more frequently met than in complex riverscapes, limiting potential biases in *Ne* estimation (Neel et al. 2013; Waples 2024b).

### 2.3 Effects of Life History Variation on *Ne* Estimation

Compared to potential impacts that affect species across several taxa, some population characteristics appear fairly restricted to one taxon. For example, the effects of the existence of plant seed banks on *Ne* estimation were identified as requiring study. Seed banks result in overlapping generations even in annual species, and models need development to examine the potential effects on *Ne* estimation. Seed banks can compensate for fluctuations in population sizes typical of some plant species by increasing *Ne* and delaying the loss of genetic diversity (Nunney 2002). Also, smolts of Pacific salmon may also reproduce at varying ages (Waples 2006). Additional limitations on *N*e estimation are associated with life‐history traits, including mating system and reproductive strategies, which influence how *Ne* varies for *Nc* and the magnitude of the *Ne*/*Nc* ratio (Gargiulo, Budde, and Heuertz, 2024). Some subsequent research has attempted to use existing literature and data to predict the direction of the bias associated with these limitations (e.g., Gargiulo et al. 2023; Neel et al. 2013; Waples 2016; Waples et al. 2013). Such predictions are challenging, as the combination of different life‐history traits would affect *Ne* and the *Ne*/*Nc* ratios possibly in contrasting directions and magnitudes.

The working groups concurred that overlapping generations, demographic fluctuations, and additional factors in long‐lived species certainly complicate *Ne* estimation. For example, in a study focusing on mammals, Pérez‐Sorribes et al. (2024) leveraged two open‐access genomic datasets from wolf populations in Minnesota and Scandinavia. These data sets represented populations with differing histories and provided known census population size *Nc* over the past 40–120 years. High‐density SNP genotypes or whole genome sequencing (WGS) data were available for testing how well GONE (Santiago et al. 2020) reconstructed real demographic changes over time, given their complex histories and certain known violations of underlying assumptions. The authors found good concordance between estimated *Ne* and trends in census size data, but the reconstruction of *Ne* highlighted the difficulty of interpreting results in spatially structured populations that had undergone demographic fluctuations.

Additional life history characteristics were identified as factors potentially influencing *Ne* estimation broadly across taxa. The propensity of some reptile lineages to have morphologically cryptic species, as in some lizards (Pinho et al. 2022), and/or overlapping generations in long‐lived species was noted. Analytical challenges are presented by the huge genome sizes of some amphibians (Liedtke et al. 2018) and the strong variation in *Ne*/*Nc* ratio reported for some amphibians (Hoban et al. 2020). Polyploidy and genome size could also present analytical challenges in plants. Integrating *Ne* estimates with other data types such as census population size, demography and population structure, and ecological data is extremely important as it allows the calibration of molecular *Ne* estimation methods and tests the sensitivity to violations of model assumptions (Mergeay et al. 2024).

### 2.4 Aspects of Datasets

The group discussed factors linked to sampling strategies known to bias *Ne* estimation, opportunities generated by new *Ne* software for use with plant data, and that very few plant species have readily available genomic resources, such as reference genomes at the chromosome level. Census sizes (*Nc*) are also mostly unavailable and difficult to determine in plants. This makes some recently developed software, such as GONE (Santiago et al. 2020), unsuitable. In contrast, resources are extensive for some charismatic mammal species (Mergeay et al. 2024; Pérez‐Sorribes et al. 2024). Similarly, Gargiulo, Decroocq, et al. (2024) explored the limitations of plant genomic datasets when estimating recent historical *Ne* using GONE. In particular, genomic datasets from non‐model species are usually derived from reduced‐representation methods, and linkage maps and reference genomes at the chromosome level are seldom available, all factors that present constraints to using GONE. The authors extracted genomic data from four plant species and showed how the accuracy and precision of *Ne* estimates changed with the extent of missing data, the number of SNPs and individuals sampled, and the lack of information about the location of SNPs on chromosomes. The latter factor, in particular, had not been previously explored with empirical data and produced a significant upward bias in the *Ne* estimation. The authors also evaluated the influence of population structure and gene pool admixture for one of the datasets, pointing out that this is influenced by the demographic history of each gene pool (e.g., recent bottlenecks). Furthermore, they evaluated the consistency of the *Ne* estimates obtained with GONE for the most recent generations and the contemporary *Ne* estimates (Santiago et al. 2024) and based on NeEstimator (Do et al. 2014). They showed a clear agreement between the estimates obtained with the latter two programs. Finally, they proposed a set of recommendations when estimating *Ne* in plants using GONE.

### 2.5 Immediate Response to Identified Issues

Through the momentum of this workshop, G‐BIKE COST Action funded targeted Short Term Scientific Missions (STSMs) of approximately a month to target specific questions in depth, thereby testing the robustness of molecular methods and helping produce guidelines for particular taxa and situations. The STSM on plants explored some of these biological and technical limitations. Results from these scientific missions have been reported (Cox, Neyrinck, and Mergeay 2024; Gargiulo, Decroocq, et al. 2024; and Pérez‐Sorribes et al. 2024).

### 2.6 Outstanding Questions to Be Addressed

Building on decades of theoretical and empirical work on *Ne*, we aimed to prepare the groundwork for testing the sensitivity of assumptions and gaining a deeper understanding of the reliability of *Ne* estimates for the latest Global Biodiversity Framework. We have presented and discussed the challenges to arrive at the optimal decision. It is crucial that scientists meticulously review all existing methods and tools for *Ne*, evaluate their current application in various species groups, and ascertain their appropriateness for future management. This comprehensive evaluation ensures that our decision‐making process is grounded in the most reliable evidence available, enabling us to make informed choices.

We lack a comprehensive synthesis of practical field applications and technological advancements in conservation genetics and genomics that would serve as the foundation for developing operational guidelines tailored to end users in conservation. Clearly, there is a need for standardized protocols, tools, and resources that can be readily implemented by practitioners. Further, ongoing collaboration between researchers, conservation practitioners, policymakers, and stakeholders is essential to ensure that guidelines are relevant, practical, and effectively disseminated. Fortunately, several studies on this challenge have been published in the past (Heuertz et al. 2023; Hoban, Bruford, et al. 2021; Holderegger et al. 2019; Kershaw et al. 2022; Lundmark et al. 2017; Taft et al. 2020). Lastly, there is a need for capacity building and training for practitioners to use the proposed tools and for scientists to be trained in the practicalities of management and constraints of real‐world situations. Fostering collaboration and knowledge exchange between practitioners and scientists could enable the advancement of refined field protocols for sampling and protocols for data analysis. Therefore, also research investment is crucial for advancing sampling protocol optimisation, and refining analytical techniques. It further includes advancements in DNA sequencing technologies, bioinformatics software, and field sampling equipment. Rigorous validation and testing of protocols in real‐world conservation scenarios are necessary to ensure their effectiveness and reliability. It involves conducting pilot studies and field trials to evaluate the performance of new protocols across different species and environmental conditions. Such investments and collaboration will enhance training programs and educational resources to equip conservation practitioners and the next generation of scientists with the knowledge and skills. Integrating refined sampling and analysis protocols into broader conservation planning frameworks ensures that genetic data are effectively utilized in decision‐making processes.

## 3 Conclusions

Despite making foundational progress in exploring current knowledge on methods of *Ne* estimation, some open questions need further investigation. A primary concern is the lack of a comprehensive synthesis of field applications and technological advancements, which would be useful as a foundation for guidelines explicitly tailored to end‐users interested in *Ne* estimation and application of Essential Biodiversity Variables (EBVs) for genetic composition (Hoban et al. 2022). Furthermore, there is an urgent need to standardize protocols, tools, and resources by using genomics data that practitioners can readily implement, thereby ensuring consistency, reliability, and comparability in their conservation strategies and population monitoring. Strengthening collaboration among various stakeholders to share expertise, data, and resources is necessary for resolving assumptions and developing strategies and solutions for data analyses. This can consolidate effective population size *Ne* as a headline indicator for the Global Biodiversity Framework and additionally contribute to practitioners' acceptance of *Ne* as an EBV (Hoban et al. 2022).

Investment in genetic and genomic approaches as well as tools is pivotal to enhancing the efficiency and accuracy of sampling and analysis. Rigorous validation and testing of new protocols in real‐world scenarios are necessary to ascertain their effectiveness and reliability across different species and environmental conditions. The provision of training programs and educational resources is essential to equip the new generation of conservation practitioners and scientists with the necessary skills and knowledge. Nevertheless, we have laid some groundwork and advocated for increased efforts to develop practical and operational guidelines for end‐users in the field of conservation genetics and genomics. Further efforts, projects, and initiatives are necessary to better address the current application and reliability of *Ne* estimates in various species groups and ascertain their appropriateness to provide practical and operational guidelines for conservation genetics and genomics end‐users.

## Conflicts of Interest

The authors declare no conflicts of interest. Joachim Mergeay, Roberta Gargiulo, and Isa‐Rita M. Russo are editorial board members of Evolutionary Applications and co‐authors of this article. To minimize bias, they were excluded from all editorial decision‐making related to this article.

## Data Availability

Data sharing is not applicable to this article as no datasets were generated or analyzed during the study at hand. *Code Availability*: No code is available to this article as no new data were analyzed or new code was created in this study.
